# Supplementary material for: Tracing the History of Hepatitis E Virus Infection in Mexico: From the Enigmatic Genotype 2 to the Current Disease Situation
Source: Viruses. 2023 Sep 12;15(9):1911. doi: 10.3390/v15091911 (PMC10536485; doi:10.3390/v15091911)
Supplement: Supplementary file 1 [file viruses-15-01911-s001.zip › viruses-2600292-supplementary.pdf]

Supplementary

Supplementary Table S1. Sequences of the HEV genome reported in Latin America.

Country of origin	Number of sequences	Genbank access	Genome region	Source	Collection (year)	Length (bp)	Genotype	Reference
Antigua and Barbuda	None							
Argentina	41	KX812460	ORF2	Swine	2005	325	--	93
		KX812461	ORF2	Swine	2005	324		93
		KX812462	ORF2	Swine	2006	355	3	93
		KX812463	ORF2	River w	2009	323	--	93
		KX812464	ORF2	Dam w	2013	325	--	93
		KX812465	ORF2	Human	2015	348	--	93
		KX812466	ORF2	Human	2014	348	--	93
		KX812467	ORF2	Human	2013	348	--	93
		KX812468	ORF2	Human	2013	348	--	93
		KX812469	ORF2	Human	2015	348	--	93
		KY511413	ORF2	River w	2013	345	--	93
		AY258006	ORF1	Swine	2003 sb	287	--	94
		AY286304	ORF2	Swine	2003 sb	148	--	94
		KJ460358	ORF1	Human	2011	252	3a	95
		KJ460359	ORF1	Human	2013	237	3i	95
		KJ460360	ORF1	Human	2013	237	3a	95
		KJ460361	ORF1	Human	2013	237	3a	95
		KF751218	ORF2	Wast w	2010	323	3	96
		KF751219	ORF2	River	2010	323	3	96
		KF751220	ORF2	Wast w	2011	319	3	96
		KF751221	ORF2	Wast w	2007	321	3	96
		KF765479	ORF1	Wast w	2010	212	3	96
		AF264009	ORF1	--	2000 sb	371	--	97
		AF264010	ORF1	--	2000 sb	371	--	97
		AF264011	ORF2	--	2000 sb	98	--	97
		AF264012	ORF2	--	2000 sb	148	--	97
		JF490047	ORF1	Human	2009	379	3	98
		JF490048	ORF1	Human	2009	255	3	98
		JF490049	ORF1	Human	2010	295	3	98
		AY548058	ORF2	Human	2004 sb	98	--	99
		AY924958	ORF2	Human	2005 sb	98	--	99
		DQ469375	ORF2	Human	2006 sb	98	--	99
		MK056268	ORF2	Swine	2018	302	--	100
		MK056269	ORF2	Swine	2018	264	--	100

		MK056270	ORF2	Swine	2018	287	--	100
		MK056271	ORF2	Swine	2018	272	--	100
		MK056272	ORF2	Swine	2018	272	--	100
		MK056273	ORF2	Swine	2018	302	--	100
		MK056274	ORF2	Swine	2018	264	--	100
		MK056275	ORF2	Swine	2018	267	--	100
		MK056276	ORF2	Swine	2018	278	--	100
		KY807297	ORF2	Water	2015	345	3	101
Bahamas	None							
Barbados	None							
Belize	None							
Bermuda	None							
Bolivia	13	JQ424437 to JQ424443	ORF2	Swine	2006	348	3i	102 and 103
		JQ424444 to JQ424449	ORF2	Human	2006	348	3e	
Brazil	154	MF981263	ORF1	Swine	2009	186	3	--
		MF981264	ORF1	Swine	2009	186	3	--
		MF981265	ORF1	Swine	2009	186	3	--
		MF981266	ORF1	Swine	2008	186	3	--
		MF981267	ORF1	Swine	2009	186	3	--
		MF981268	ORF1	Swine	2009	186	3	--
		MF981269	ORF1	Swine	2009	186	3	--
		MF981270	ORF2	Swine	2009	257	3	--
		MF981271	ORF2	Swine	2009	257	3	--
		MF981272	ORF2	Swine	2009	257	3	--
		MG573667	ORF2	Monkey	2017	302	3	104
		MZ061632	ORF1	Human	2016	255	3	105
		MZ061633	ORF1	Human	2017	255	3	105
		MZ061634	ORF1	Human	2017	255	3	105
		MZ061635	ORF1	Human	2017	255	3	105
		MZ061636	ORF1	Human	2018	255	3	105
		MZ061637	ORF1	Human	2018	255	3	105
		OP485092	ORF1	Capybara	2019	442	3	106
		OQ446059	ORF2	Human	2006	1036	3	107
		MH664123	ORF2	Swine	2017	304	3	108
		MH664124	Complete	Swine	2017	7233	3	108
		KX757780	ORF1	Human	2015	242	3	--
		KX757781	ORF2	Human	2015	304	3	--
		KX770298	ORF1	Human	2015	217	3	--
		KY765017 to KY765021	ORF2	Swine	2015	303		109
		MF438128	ORF1	Swine	2014	207	3c	110
		MF438129	ORF1	Swine	2014	207	3c	110

		MF438130 to MF438133	ORF1	Swine	2014	207	3h	110
		MF438134	ORF2	Swine	2014	304	3c	110
		MF438135	ORF2	Swine	2014	304	3b	110
		JN983192	ORF2	Swine	2010	323	3	111
		JN983193	ORF2	Swine	2010	338	3c	111
		JN983194	ORF2	Swine	2010	336	3f	111
		JN983195	ORF2	Swine	2010	336	3f	111
		JN983196	ORF2	Swine	2010	249	3f	111
		JN983197	ORF2	Swine	2010	336	3f	111
		JN983198	ORF2	Swine	2010	345	3c	111
		JN983199 to JN983206	ORF1	Swine	2010	266	3c	111
		JN983207	ORF1	Swine	2010	249	3c	111
		JN983208	ORF1	Swine	2010	266	3c	111
		JN983209	ORF1	Swine	2010	266	3c	111
		JN983210	ORF1	Swine	2010	266	3f	111
		JN983211	ORF1	Swine	2010	266	3c	111
		JN983212	ORF1	Swine	2010	266	3c	111
		KU888659 to KU888663	ORF1	Swine	2015	230	3	112
		KU888664	ORF1	Swine	2011	288	3	--
		KU888665	ORF1	Swine	2011	288	3	--
		KX578263	ORF1	Swine	2013	245	3	112
		KX578264	ORF1	Monkey	2015	242	3	113
		KX578265	ORF1	Monkey	2015	242	3	113
		KX578266	ORF1	Monkey	2015	242	3	113
		KX578267	ORF2	Swine	2013	304	3	112
		KX578268	ORF2	Monkey	2015	304	3	113
		KX578269	ORF2	Monkey	2015	304	3	113
		KX578270	ORF2	Monkey	2015	304	3	113
		KY907039 to KY907051	ORF1	Swine	2012	266	3	114
		KY907052 to KY907064	ORF2	Swine	2012	423	3	114
		JX123025	ORF2	Swine	2010	304	3	115
		JX123026	ORF2	Swine	2010	304	3	115
		JX173928	ORF2	Human	2012sb	304	3	116
		JX173929	ORF2	Human	2012sb	304	3	116
		JX173930	ORF2	Human	2012sb	304	3	116
		KF719311	ORF2	Swine	2012	342	3	--
		KF719312	ORF2	Swine	2012	336	3	--
		KF719313	ORF2	Swine	2012	336	3	--

		KF719314	ORF2	Swine	2012	336	3	--
		KF719315	ORF2	Swine	2012	336	3	--
		KF719316	ORF2	Swine	2012	306	3	--
		KC549910 to KC549917	ORF2	Swine	2011	304	3	117
		KF152884	ORF2	Human	2011	304	3b	118
		KM502569	ORF2	Human	2009	3004	3b	118
		KP966825	ORF2	Swine	2014	304	3b	119
		KP966826	ORF2	Swine	2014	304	3b	119
		KP966827	ORF2	Swine	2014	304	3b	119
		KP966828	ORF1	Swine	2014	204	3b	119
		KP966829	ORF1	Swine	2014	242	3b	119
		EF491206	ORF2	Swine	2003	1762	3	--
		GQ421465	ORF1	Human	2009 sb	242	3	120
		HM154537	ORF1	Swine	2009	240	3	121
		HM154538	ORF1	Swine	2008	240	3	121
		HM154539 to HM154542	ORF1	Swine	2008	340	3	121
		HM154543 to HM154547	ORF2	Swine	2008	141	3	121
		JN166093	ORF1	Swine	2010	243	3	115
		JN166094	ORF1	Swine	2010	242	3	115
		JN190065 to JN190070	ORF2	Swine	2009	304	3	122
		JN190071	ORF2	Swine	2009	242	3	122
		JN190072	ORF2	Swine	2009	242	3	122
		EF591852	ORF1	Swine	2007 sb	237	3	123
		EF591853	ORF1	Swine	2007 sb	237	3	123
		EF591854	ORF2	Swine	2007 sb	303	3	123
		EF591855	ORF2	Swine	2007 sb	303	3	123
		EF591856	ORF2	Swine	2007 sb	303	3	123
Chile	None							
Colombia	74	KU639880 to KU639890	ORF2	Swine	2012	815	3	124
		KU639891 to KU639953	ORF2	Swine	2012	298	3	124
Costa Rica	12	DQ677372 to DQ677377	ORF2	Swine	2006 sb	212	3	125
		DQ677378	ORF2	Swine	2006sb	179	3	125
		DQ677379	ORF2	Swine	2006 sb	212	3	125
		DQ677380	ORF2	Swine	2006 sb	212	3	125

		DQ677381	ORF2	Swine	2006 sb	209	3	125
		DQ677382	ORF2	Swine	2006 sb	212	3	125
		DQ677383	ORF2	Swine	2006 sb	212	3	125
Cuba	55	EU284748	ORF2	Human	2007 sb	354	1	126
		EU284749	ORF2	Human	2007 sb	354	1	126
		EF493155	ORF1	Human	2007 sb	307	1	126
		EU165494 to EU165504	ORF1	Human	2007 sb	241	1	126
		JF937673 to JF937678	ORF1	Swine	2007	201	3	--
		HQ446873 to HQ446878	ORF1	Human	2008	279	3	--
		FJ769237	ORF1	Human	2007	270	3	127
		FJ769238	ORF1	Human	2007	260	3	127
		HQ446879	ORF2	Human	2008	149	3	--
		LM654183	ORF2	Dolphin	2014	167	3	128
		LM654184	ORF2	Dolphin	2014	167	3	128
		KP096349	ORF1	Dolphin	2014	289	3	128
		KM065571	ORF1	Dolphin	2007	298	3b	128
		KM102180	ORF2	Dolphin	2007	322	3	128
		FJ948818	ORF1	Human	2007	240	3	--
		FJ948819	ORF1	Human	2007	246	3	--
		LN849722	ORF2	Human	2013	125	3	--
		LN849723	ORF2	Human	2013	125	3	--
		OM867876	ORF2	Swine	2016	336	3	129
		OM867877	ORF2	Swine	2016	336	3	129
		OM867878	ORF2	Swine	2007	291	3	129
		OM867879 to OM867885	ORF2	Swine	2007	336	3	129
		OM867886	ORF2	Swine	2016	336	3	129
		OM867887	ORF2	Swine	2016		3	129
Dominican Republic	None							
Ecuador	None							
El Salvador	None							
Grenada	None							
Guatemala	None							
Guyana	None							
Haiti	3	AF047864	ORF2	Human	1998 sb	448	--	130
		AF047865	ORF1	Human	1998 sb	233	--	130

		AF047866	ORF1	Human	1998 sb	137	--	130
Honduras	None							
Jamaica	None							
Mexico	64	AF521654	ORF1	Swine	2002 sb	257	--	--
		AY858902 to AY858937	ORF2	Swine	2004 sb	304	3	25
		MF116409	ORF2	Swine	2016	304	3a	--
		MF116410	ORF2	Swine	2016	303	3a	--
		MF116411	ORF2	Swine	2016	304	3a	--
		MF116412	ORF2	Swine	2016	304	3a	--
		MG570165	ORF2	Human	2012	212	3	29
		MG570166	ORF2	Human	2013	212	3	29
		MG833836	Complete	Swine	2016	7251	3a	89
		MG980615	ORF1/2	Swine	2016	4775	3a	89
		MH003296	ORF2	Swine	2016	1473	3a	89
		MH844063	ORF1	Human	2011	240	3	28
		MH844064	ORF1	Human	2011	240	3	28
		MH844065	ORF1	Human	2013	222	3	28
		MH844066	ORF1	Human	2015	222	3	28
		MH844067	ORF!	Human	2016	222	3	28
		MH844068	ORF1	Human	2015	225	3	28
		MH844069	ORF1	Human	2013	225	3	28
		MH844070	ORF1	Human	2011	240	3	28
		MH844071	ORF1	Human	2015	240	3	28
		MH844072	ORF1	Human	2011	195	3	28
		MH844073	ORF1	Human	2016	240	3	28
		MH844074	ORF1	Human	2016	240	3	28
		MF039701	ORF2	Human	2017 sb	322	1	27
		MF039702	ORF2	Human	2017 sb	323	1	27
		MF039703	ORF2	Human	2017 sb	701	1	27
		MF039704	ORF2	Human	2017 sb	318	1	27
		MF039705	ORF2	Human	2017 sb	701	1	27
		KX578717	Complete	Human	1986	7159	2a	40
Nicaragua	None							
Panama	1	JQ001745	RNA pol	Bat	2009	127	--	131
Paraguay	None							
Peru	None							
San Vincent Grenadines	None							
St. Lucia	None							
Suriname	None							

Trinity and Tobago	None							
Uruguay	54	MG182432	ORF2	Swine	2017 sb	272	--	--
		MG182433	ORF2	Swine	2017 sb	272	--	--
		MG182434	ORF2	Swine	2017 sb	298	--	--
		MW59686	Complete	Human	2018	7229	3	--
		MT774175	ORF1	Human	2017	1446	3	--
		MF590059	ORF1	Swine	2015	215	3	132
		MF590060	ORF1	Swine	2015	215	3	132
		MF590061	ORF1	Swine	2015	215	3	132
		MG182431	ORF1	Human	2016	214	3	--
		MG432489	ORF1	Human	2017 sb	159	3	--
		MG432490	ORF1	Human	2017 sb	159	3	--
		MT430885	ORF1	Human	2017	210	3	133
		MT430886	ORF1	Human	2017	214	3	133
		MT497901	ORF2	Peccary	2018	326	3	134
		MT497902	ORF2	Human	2018	354	3	135
		MT497903	ORF2	Human	2017	330	3	135
		KF679999	ORF1	Human	2010	282	3	136
		KF680000	ORF1	Human	2010	282	3	136
		KF680001	ORF1	Human	2013	214	3	136
		JX880196 to JX880203	ORF2	Human	2012 sb	768	3	137
		JX880204	ORF1	Human	2010 sb	480	3	137
		JX880205	ORF1	Human	2012 sb	481	3	137
		JX880206	ORF1	Human	2012 sb	477	3	137
		JX880207	ORF1	Human	2012 sb	481	3	137
		JX880208	ORF1	Human	2012 sb	482	3	137
		JX880209	ORF1	Human	2012 sb	482	3	137
		JX880210	ORF1	Human	2012 sb	482	3	137
		JX880211 to JX880216	ORF1	Human	2012 sb	210	3	137
		JX880217	ORF1	Human	2012 sb	209	3	137
		JX880218	ORF1	Human	2012 sb	210	3	137
		JX880219	ORF1	Human	2012 sb	207	3	137
		JX880220	ORF1	Human	2012 sb	210	3	137
		KC189822	ORF2	Human	2012 sb	844	3	137
		HM775425	ORF2	Human	2010 sb	76	3	138
		HM775426	ORF2	Human	2010 sb	76	3	138
		HM765427	ORF2	Human	2010 sb	76	3	138
		HM765428	ORF2	Human	2010 sb	75	3	138
		HM765429	ORF2	Human	2010 sb	76	3	138
		HQ724532	ORF3	Human	2010 sb	75	3	138
Venezuela	3	HM366940	ORF1	Human	2008	329	3	139
		HM366941	ORF1	Human	2008	329	1	139

		HM366942	ORF1	Human	2008	331	1	139

Sb= Submission year
